# Estimating Compressive Strength of Concrete Containing Untreated Coal Waste Aggregates Using Ultrasonic Pulse Velocity

**DOI:** 10.3390/ma14030647

**Published:** 2021-01-31

**Authors:** Mahmood Karimaei, Farshad Dabbaghi, Mehdi Dehestani, Maria Rashidi

**Affiliations:** 1 Faculty of Civil Engineering, Babol Noshirvani University of Technology, Babol 47148-71167, Iran; mahmood.karimaei@gmail.com (M.K.); dehestani@nit.ac.ir (M.D.); 2 Centre for Infrastructure Engineering, Western Sydney University, Penrith, NSW 2751, Australia; m.rashidi@westernsydney.edu.au

**Keywords:** concrete, untreated coal waste, fine recycled aggregates, coarse recycled aggregates, recycling, ultrasonic pulse velocity (UPV), compressive strength

## Abstract

In recent years, the overuse and exploitation of coal resources as fuel in industry has caused many environmental problems as well as changes in the ecosystem. One way to address this issue is to recycle these materials as an alternative to aggregates in concrete. Recently, non-destructive tests have also been considered by the researchers in this field. As there is limited work on the evaluation of the compressive strength of concrete containing coal waste using non-destructive tests, the current study aims to estimate the compressive strength of concrete containing untreated coal waste aggregates using the ultrasonic pulse velocity (UPV) technique as a non-destructive testing approach. For this purpose, various concrete parameters such as the compressive strength and UPV were investigated at different ages of concrete with different volume replacements of coarse and fine aggregates with coal waste. The test results indicate that 5% volume replacement of natural aggregates with untreated coal waste improves the average compressive strength and UPV of the concrete mixes by 6 and 1.2%, respectively. However, these parameters are significantly reduced by increasing the coal waste replacement level up to 25%. Furthermore, a general exponential relationship was established between the compressive strength and the UPV associated with the entire tested concrete specimens with different volume replacement levels of coal waste at different ages. The proposed relationship demonstrates a good correlation with the experimental results.

## 1. Introduction

The production of global solid waste is persistently accelerating with the advancement of industry and emerging technology applications, as well as the increase in the human population. In this regard, coal is considered as one of the most essential sources of energy throughout the world, the extraction and exploitation of which lead to the production of waste materials [1,2]. Coal is one of the most abundant resources used to produce energy. Coal production across the world is about 5.5 billion tons per year, and the volume produced in Iran reaches about 310 million tons per year. In general, there are three ways to dispose of such waste in nature: landfills, incineration, and recycling. The latter one has become a potential solution for the disposal management of such waste [3]. In this respect, researchers have made great efforts in the field of waste recycling and its reuse in the civil engineering discipline [4,5,6]. An efficient method for recycling solid waste is to use them in concrete; a practice that not only prevents the direct release of the solid wastes into the environment, but is also able to lower the consumption of quarried aggregates [5,7]. Over the past years, studies have been carried out on the recycling of various materials such as scrap tires, polyethylene terephthalate (PET), concrete, glass, etc. in various types of concrete. On the one hand, the use of coal waste as a partial replacement of aggregates in concrete reduces the depletion of natural resources and mitigates environmental hazards. On the other hand, coal waste can show similar properties to cement due to the presence of silica and alumina in its composition, and thus improve the mechanical and physical properties of concrete. Scholars have conducted extensive research on the use of coal waste as a volume replacement of natural aggregates in concrete. In a study, Cassiano et al. [8] used coal waste as an alternative to fine aggregates in concrete. They observed that replacing 25 and 50% of fines with coal waste improved the mechanical properties of concrete after 28 days. Hesami et al. [9] examined the effect of coal waste on the strength characteristics of roller concrete. The results indicated that replacing 5% of coal powder improved the strength properties of concrete for up to 90 days. However, adding 10 and 20% of coal powder reduced the compressive and tensile strengths of roller concrete at different ages. Karimaie et al. [10] investigated the mechanical specifications of concrete with coal waste replacing aggregates. It was concluded that the effect of coal waste on concrete at 5% of volume addition improved the mechanical properties of concrete. The substitution of aggregates with coal waste increased the average compressive and flexural strengths by about 3–7 and 5–8%, respectively. In another study, Karimpour [3] evaluated the effect of untreated coal waste as an alternative to coarse and fine aggregates on the mechanical properties of concrete. The experimental results showed that adding 5 vol.% of coal waste enhanced the mechanical properties of concrete, while a further increase of coal reduced such properties.

On account of the uncertainties present in the strength assessment of concrete specimens, as well as the localized damages induced by weathering, fire, and chemical attacks, civil engineers are increasingly demanding advanced and reliable methods for the evaluation and quality control of the concrete. In this respect, non-destructive test methods have attracted the attention of civil engineers due to the reduction in testing time, convenience of testing, and low cost compared to destructive approaches. Besides, these procedures have demonstrated promising results in inspecting and evaluating the quality of existing concrete structures [11,12,13,14,15]. The ultrasonic pulse velocity (UPV) technique is a non-destructive test method of concrete based on the calculation of ultrasonic pulse transmission speed within concrete. It has been broadly implemented to estimate various specifications and integrity of concrete structures [13,14]. Basically, the characteristics of concrete, including strength, elastic modulus, porosity, depth of surface cracks, defects, and damages caused by chemical attacks and fire, can be evaluated using UPV [5,16]. The speed of the ultrasonic pulse is affected by many aspects, such as cement type and content, age of concrete, water-to-cement ratio, size and type of aggregates, curing method, temperature of the measuring medium, and the length of measuring distance [15,17,18]. In recent years, several researchers have focused attention on the ultrasonic pulses in different fields of study. Washer et al. [19], by examining the experimental results of longitudinal and shear pulse velocities of cylindrical and cubic concrete specimens, concluded that the velocity of the pulse is dependent on the presence of fibers, curing method, modulus of elasticity, and density of concrete. They also proposed that the propagation of ultrasonic pulses at high frequencies as high as 1 MHz or higher can be launched and received. It was also stated that increasing the test frequency slightly increases both the longitudinal and shear pulse velocities. Bogas et al. [18] assessed the compressive strength of lightweight concrete using UPV. In their research, the pulse velocity of specimens with varying water-to-cement ratios, type and volume of aggregates, type of pozzolan, and different ages of concrete was investigated. They could provide pulse velocity ranges according to the type of variable, and then developed a relationship between compressive strength and UPV in the form of an exponential function. Thus, a simplified expression for estimating the compressive strength was recommended, regardless of the concrete type and composition. Despite the many studies on the estimation of concrete compressive strength using UPV [18,19,20,21,22], there is little research in the literature on the relationship between compressive strength and UPV of concrete containing untreated coal waste. Therefore, this study aims to examine the performance of the UPV method in estimating the compressive strength and the quality of specimens containing untreated coal waste. Accordingly, the concrete specimens were made in 11 experimental groups, and the parameters of compressive strength and UPV at different ages, and with different percentages of coal waste substituting coarse and fine aggregates, were investigated. The UPV test was performed on specimens after 7, 14, and 28 days of fabrication.

## 2. Experimental Program

### 2.1. Materials and Specimens

**Figure 1:**
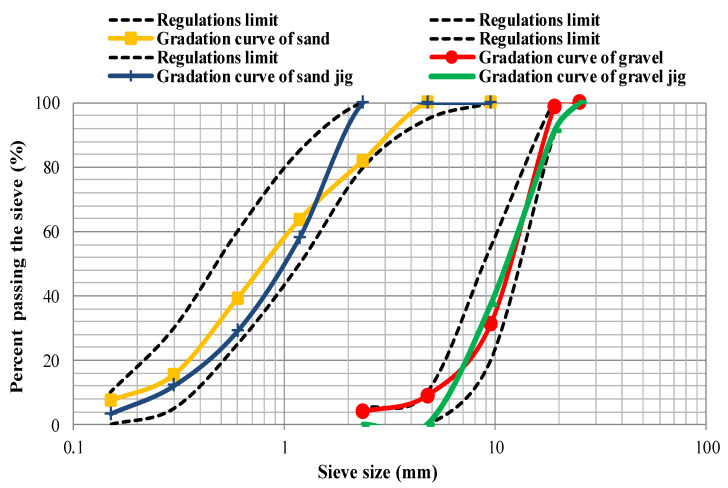
Gradation curves of natural and coal waste aggregates.

**Figure 2:**
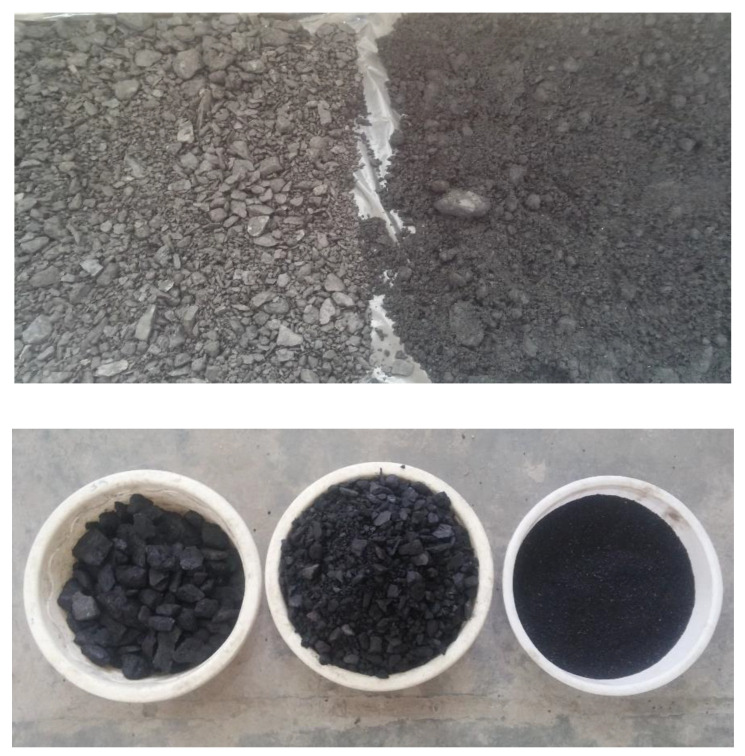
Untreated coal waste.

In this study, crushed stone was used as coarse aggregate with a maximum nominal size of 12.5 mm. The crushed sand was also used as fines with a fineness modulus of 2.92 and a maximum grain size of 4.75 mm. The gradation curves of coarse and fine aggregates are illustrated in Figure 1, following the requirements of ASTM C33 [23]. Furthermore, the properties of the aggregates are reported in Table 1. In addition, untreated coal waste was utilized as a recycled material replacing coarse and fine aggregates, which was obtained from a Jig concentrator located in the Central Alborz Coal Preparation Plant, Zirab Northern Iran. Minerals extracted from mining are typically infused with a significant amount of impurities, the presence of which mostly reduces the mineral grade to such an extent that it will not have the necessary economic value without processing and upgrading operations. Jig is one of the gravity upgrading equipment. Despite the implementation of various gravity concentrators today, Jig has a special role in coal washing due to its high washing capacity and low operating costs. Untreated coal waste has a dry unit weight of 1.24 g/cm^3^, compressive strength of about 25 MPa, and fineness modulus of about 2.9. The chemical properties and the gradation curves of both replacement levels of waste coal used in this study are presented in Table 2 and Figure 1, respectively. Moreover, Figure 2 shows an image of the coal waste utilized. In addition, Type 2 Portland cement was purchased from Mazandaran Cement Factory, Northern Iran. The chemical, physical, and mechanical characteristics of cement are presented in Table 3.

In this study, a total of 99 cubes with 100-mm dimensions were fabricated in 11 experimental groups to evaluate the influence of coal waste on compressive strength and pulse velocity at 7, 14, and 28 days of concrete age. The test results are the average of three similar specimens made from the same concrete and tested at the same age.

### 2.2. Mixing Proportions and Specimen Preparation

The mix design proportions for one cubic meter of concrete, according to the ACI 211.1R specifications [24], are presented in Table 4. As reported in this table, the ratio of water-to-cementitious materials used for the entire mixes was taken as 0.55. In addition, the coal waste was considered as a variable in five different volumes of 5, 10, 15, 20, and 25% in the concrete mixing procedure.

In order to prepare the concrete specimens, the gravel and sand were first mixed with coal waste aggregates in the mixer for 1 min. The cement was then added and mixed for another 1 min. Afterward, water was gradually added to the mixture in 30 s, and the mixture was stirred for 2 min. After the mixing process, the slump test of concrete mixtures was measured according to ASTM C143 [25] to specify the workability of freshly made concrete, the values of which are given in Table 4. Then, the fresh concrete was poured into 100 mm cubic molds in two layers, each compacted by 25 strokes of a tamping rod. A vibrating table was used for compaction and the removal of air bubbles. The cubic specimens were demolded after 24 h and cured under ASTM C192 [26] for at least 28 days.

The notation of the mixtures, listed in Table 4, was carried out based on the test variables. Accordingly, mix scheme 1 shows the concrete with no coal waste (CS) (control specimen). Furthermore, gravel is replaced with coal waste in the G-J-S (gravel jig specimen) group, and sand is substituted with coal waste aggregates in the S-J-S (sand jig specimen) group.

### 2.3. Testing Procedure

**Figure 3:**
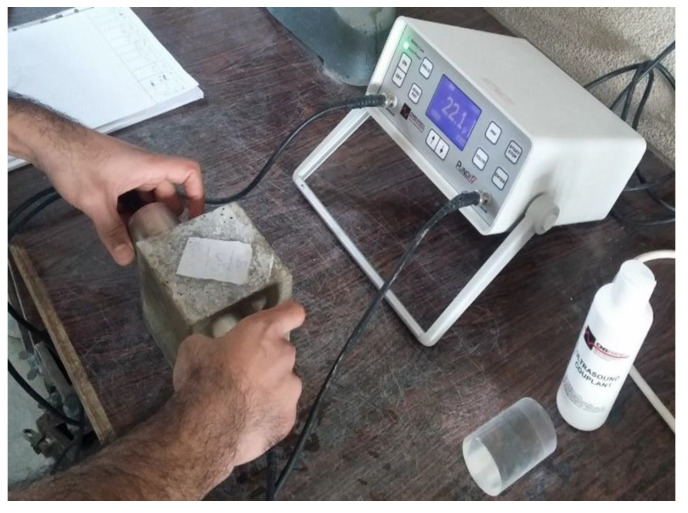
Pundit testing tool.

**Figure 4:**
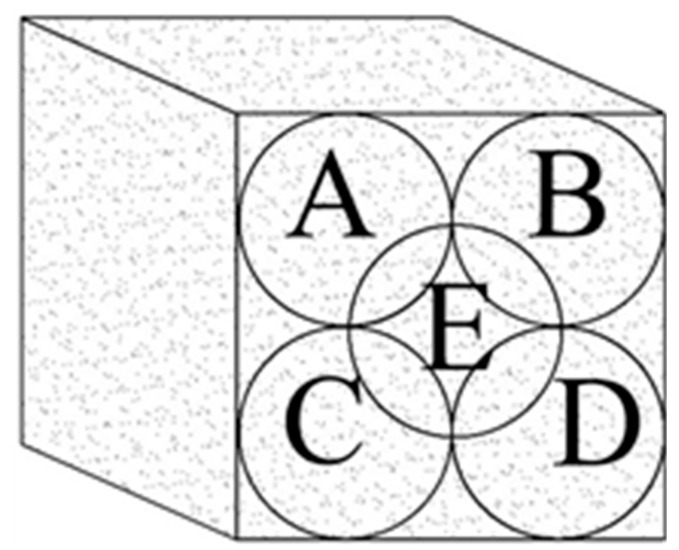
Testing point locations.

An ultrasonic non-destructive electronic machine with an accuracy of 0.1 µs was used to measure the UPV complying with ASTM C597 [27]. The non-destructive testing of specimens at the ages of 7, 14, and 28 days was performed to specify the transmission time of pulse through a direct procedure with a Pundit (PC 1012) testing tool, as shown in Figure 3. A transducer with an oscillation frequency of 54 kHz, transit time accuracy of ±1%, and distance accuracy of ±2% was used. Refractory grease was added in all tests to couple the transducers onto the even surface of concrete. Five points were tested on each specimen by changing the location of transducers on the opposite sides of the specimen, the average of which was reported as a result. Figure 4 presents the schematic of measuring points on tested specimens.

Once the transmission time test results were determined, the ultrasonic pulse transmission speed in concrete was obtained by dividing the transmission distance (distance between the measuring points) by the transmission time. Subsequently, the compression test was conducted on cubes at a loading rate of 0.25 MPa/s, conforming to the BS EN 12,390 specifications [28]. The compressive strength of all specimens was then calculated in MPa.

## 3. Results

### 3.1. Test Results and Discussion

In this section, the relationship between the effective parameters, including the specimen age, coal waste content, and the replacement scheme on the pulse velocity and compressive strength, is discussed. The results of compressive strength and UPV of concrete specimens after 7, 14, and 28 days are presented in Table 5.

### 3.2. The Effect of Type and Amount of Coal Waste on UPV

**Figure 5:**
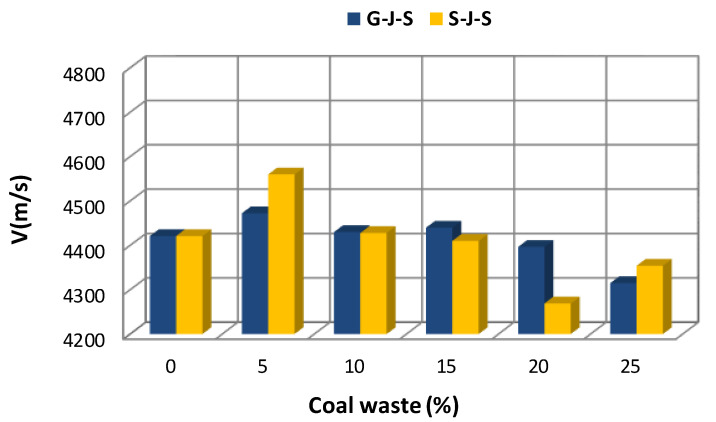
Effect of replacing aggregates with coal waste at the age of 7 days.

**Figure 6:**
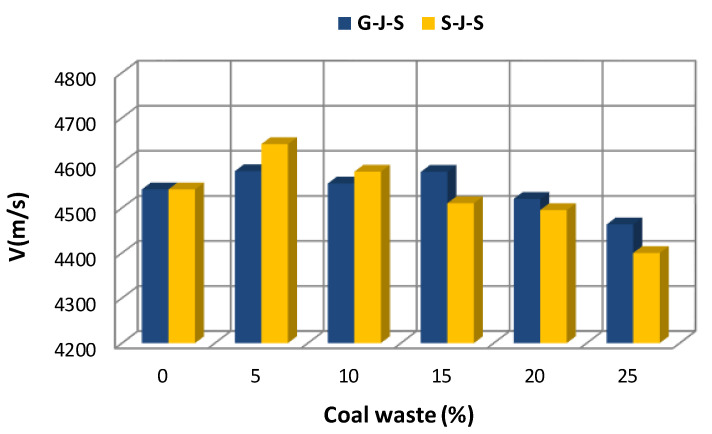
Effect of replacing aggregates with coal waste at the age of 14 days.

**Figure 7:**
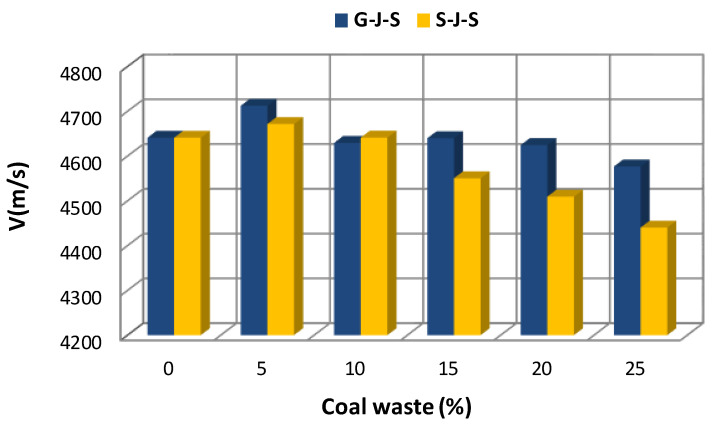
Effect of replacing aggregates with coal waste at the age of 28 days.

Figure 5, Figure 6 and Figure 7 illustrate the effect of the type and amount of coal waste on the UPV of concrete cubes at the ages of 7, 14, and 28 days, respectively. Based on these figures, it is discerned that the UPV is significantly decreased with increasing coal waste replacing coarse and fine aggregates. This can be attributed to higher porosity and lower integrity of concrete in this group. In other respect, the reduction in UPV is associated with small pores within the coal waste structure that induce a weaker interfacial transition zone (ITZ) in comparison with the control specimen (with no coal waste). Therefore, these pores and microcracks reduce pulse velocity. Nevertheless, adding 5% of coal waste has the greatest effect on increasing the pulse velocity. In addition, according to Figure 5, Figure 6 and Figure 7 and Table 5, it is observed that the maximum pulse velocity for the 28-day specimen with 5% of coal waste replacing coarse aggregates is 4712.4 m/s, which is 1.53% higher compared to the control specimen at that age. In contrast, the minimum velocity corresponds to the 7-day specimen with 25% of coal waste replacing fine aggregates, marking 4268.9 m/s. This value is even lower than that of the control specimen of the same age with a pulse velocity of 4421 m/s. Figure 6 shows the effect of coal waste on the UPV of concrete after 14 days of curing. Exchanging 5% of fines with coal waste has the highest effect on increasing the pulse velocity by 4641.7 m/s, while the minimum velocity shows 4400 m/s, belonging to the concrete containing 25% of coal waste aggregates. The effect of coal waste on the UPV of concrete at 28 days is shown in Figure 7. Accordingly, adding 5% of coal waste replacing coarse and fine aggregates increases the pulse velocity. In this sense, substituting coarse aggregates with untreated coal waste demonstrates the greatest influence on increasing the pulse velocity compared to fine aggregates replacement. However, the pulse velocity of concrete at the age of 28 days is reduced by 1.4–4.3% by replacing more than 5% of aggregates with coal waste, compared to the control specimen.

### 3.3. The Effect of Type and Amount of Coal Waste on Compressive Strength

**Figure 8:**
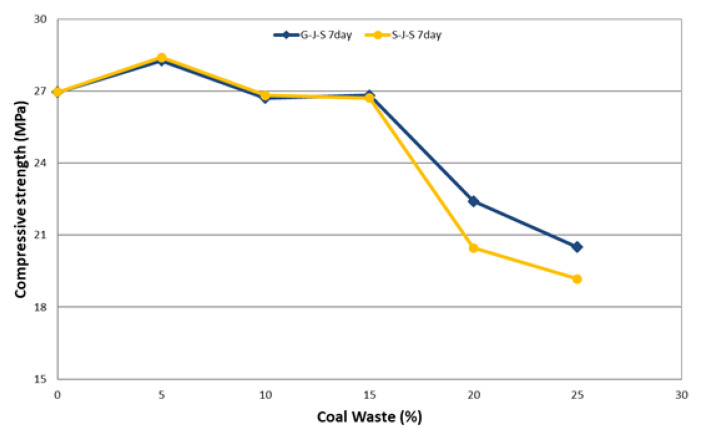
Effect of type and amount of coal waste on the compressive strength after 7 days.

**Figure 9:**
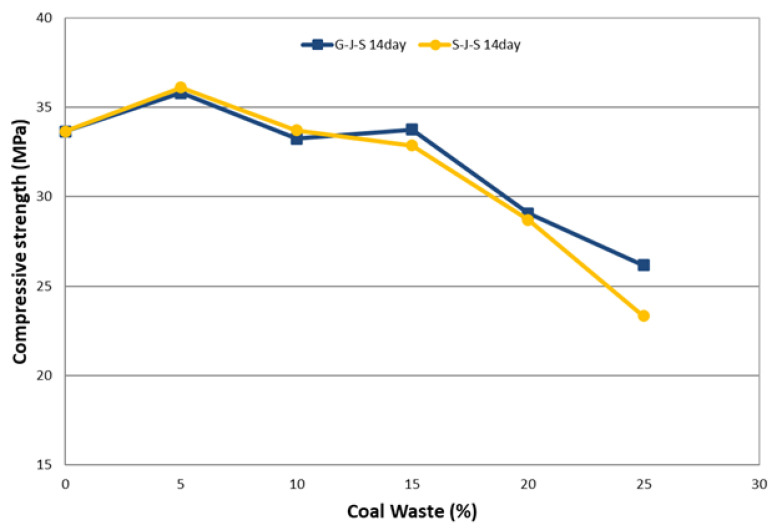
Effect of type and amount of coal waste on the compressive strength after 14 days.

**Figure 10:**
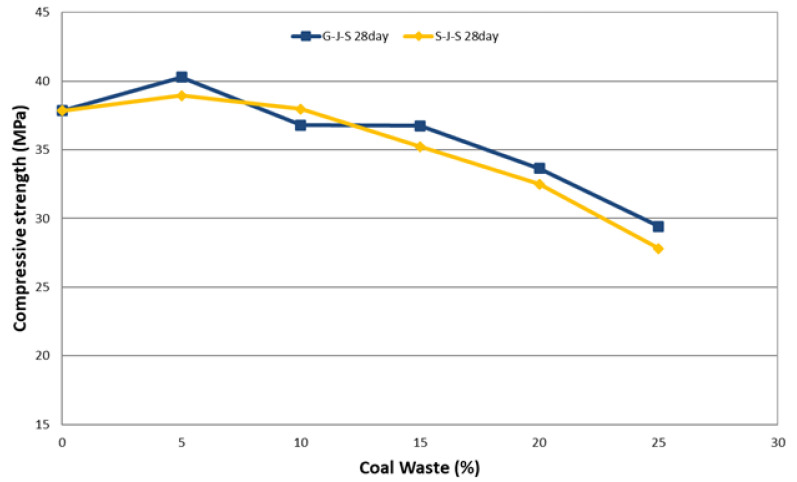
Effect of type and amount of coal waste on the compressive strength after 28 days.

The effect of the type and amount of coal waste on compressive strength of concrete at different ages is presented in Table 5 and Figure 8, Figure 9 and Figure 10. The declining trend of compressive strength is seen by increasing the replacement level of aggregates with coal waste. The reason for such reduction is the presence of a porous network within the coal waste aggregates and the absence of hard cement paste–aggregate bond, thus inducing a weaker (Interfacial Transition Zone) ITZ as to the control specimen. Therefore, when concrete is exposed to compression loading, cracking initiates rapidly around the recycled aggregates (coal waste), reducing the compressive strength. Another reason could be the compressive strength of untreated coal aggregates, being one-third of the natural companions, accelerates the failure of the specimen by increasing the replacement content [3,10]. Nonetheless, adding 5% of recycled aggregates improves the compressive strength by about 5.5% compared to the control concrete. Thereby, 5% of coal waste replacement can be considered as the optimum amount of recycled aggregates in this investigation, which is observable at all ages. In this respect, the compressive strength of specimen with 5% of coal waste is increased by about 5.9% compared to that without recycled aggregates. However, the compressive strength of mixes with 10, 15, 20, and 25% of coarse coal waste aggregates (coal waste replacing natural coarse aggregates) is decreased by 1.7, 1.1, 13.9, and 22.8%, respectively, in comparison with the control specimen. Furthermore, the compressive strength of the specimen containing 5% of fine coal waste aggregates (coal waste replacing natural fine aggregates) is increased by about 5.2% compared to the control specimen. By contrast, the compressive strength of concrete specimens containing 10, 15, 20, and 25% of fine coal waste aggregates is decreased by 0.5, 3.4, 17.6, and 28.7%, respectively, compared to the control concrete. Consequently, the use of coal waste as the volume replacement of sand, as opposed to gravel, has a more pronounced impact on decreasing the compressive strength of specimens.

### 3.4. The Effect of Concrete Age on UPV and Compressive Strength

**Figure 11:**
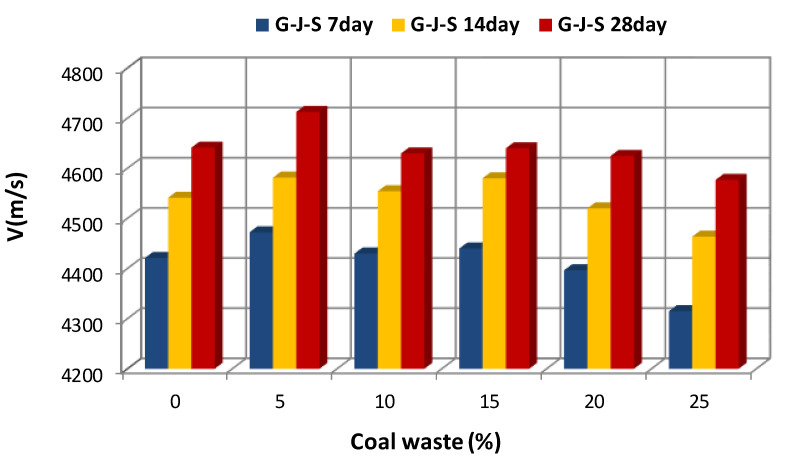
The effect of concrete age on the UPV of the G-J-S group.

**Figure 12:**
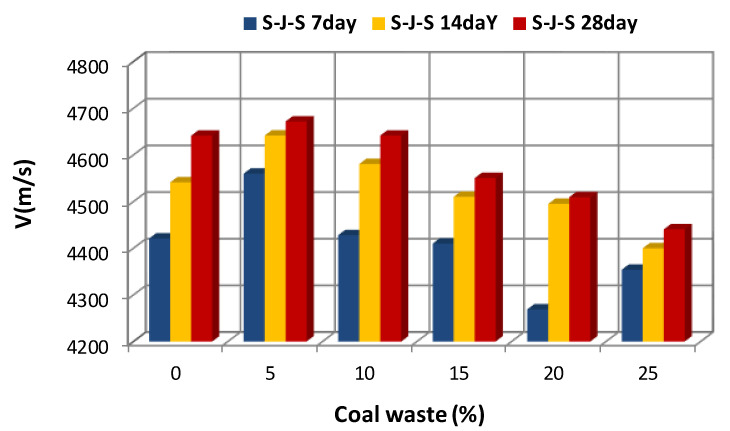
The effect of concrete age on the UPV of the S-J-S group.

The results of the pulse velocity test for all specimens after 7, 14, and 28 days of curing are presented in Figure 11 and Figure 12. Based on these figures, it is observed that the pulse velocity is increased with increasing the age of concrete, which is likely due to the removal of capillary porosity and microcracks within the cement paste, as well as the evolution of hydration process as the concrete ages [10]. According to the literature, improvement in the cement matrix has a considerable effect on increasing the strength of cement and concrete. Figure 11 shows the effect of concrete age on the UPV of the G-J-S group. It is seen that by replacing 5% of coarse aggregates with coal waste at different ages, the pulse velocity has the highest value compared to other specimens. However, the lowest pulse velocity at different ages belongs to concrete with 25% of coal waste. Likewise, the effect of concrete age on the UPV of the S-J-S group is shown in Figure 12. By replacing 5% of fine aggregates with coal waste, the pulse velocity has the highest measure at different ages, while the lowest value belongs to concrete with 20% of fine coal waste aggregates at 7 days. The lowest pulse velocities at 14 and 28 days are also associated with concrete containing 25% of fine coal waste aggregates.

**Figure 13:**
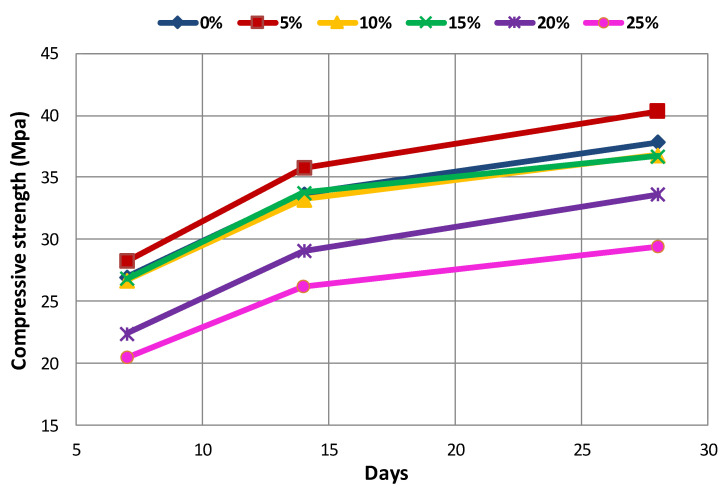
The effect of concrete age on the compressive strength of G-J-S group.

**Figure 14:**
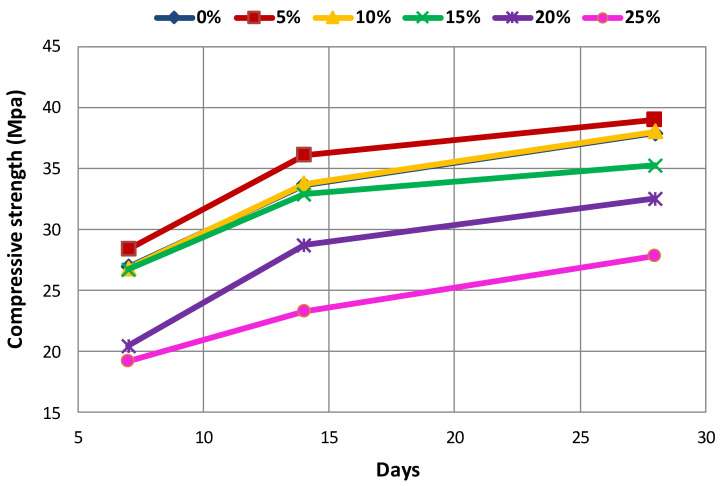
The effect of concrete age on the compressive strength of S-J-S group.

The relationship between the concrete age and the compressive strength can be seen separately in Figure 13 and Figure 14 for both replacement series. The results indicate that the strength gain has an increasing trend, which will even improve over time. According to these two figures, it can be seen that the compressive strength increases with increasing the age of the concrete, which is due to the evolution of the hydration process and the elimination of concrete porosity. Figure 13 and Figure 14 illustrate the effect of concrete age on the compressive strength in the G-J-S and S-J-S groups, respectively. As natural aggregates are replaced with coal waste by 5%, the compressive strength is improved. However, as more coal waste is used in the mix design, the compressive strength is reduced at different ages. According to the figures, the maximum compressive strength for the 28-day specimen with a replacement level of 5% of coarse coal waste is 40.3 MPa, which is approximately 6% higher compared to the control specimen at the same age. On the contrary, the lowest compressive strength for the 7-day specimen is 19.2 MPa with a 25% replacement of fine coal waste, indicating an approximately 29% reduction compared to the control specimen at that age.

### 3.5. Comparing Compressive Strength and UPV

**Figure 15:**
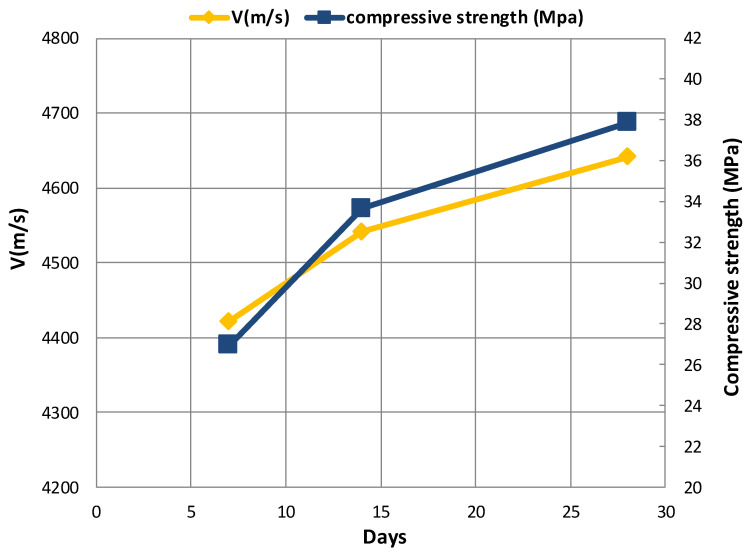
Comparison of UPV and compressive strength in the control specimen (CS).

**Figure 16:**
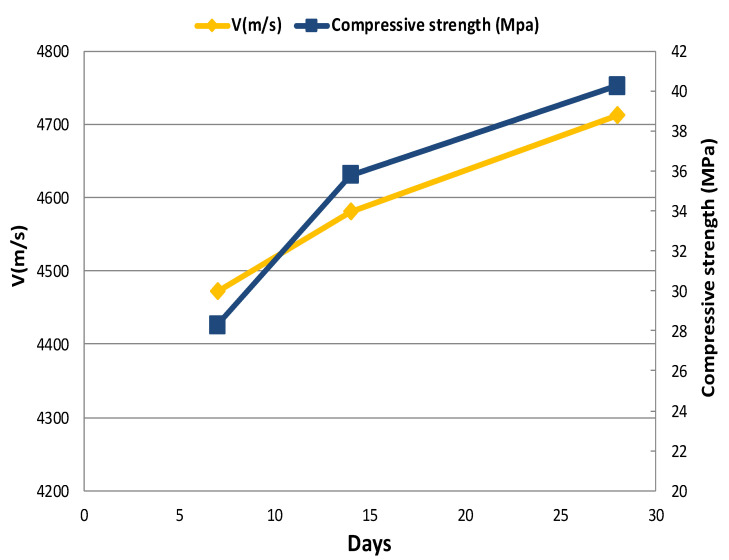
Comparison of UPV and compressive strength at 5% replacement of gravel (G-J-S).

**Figure 17:**
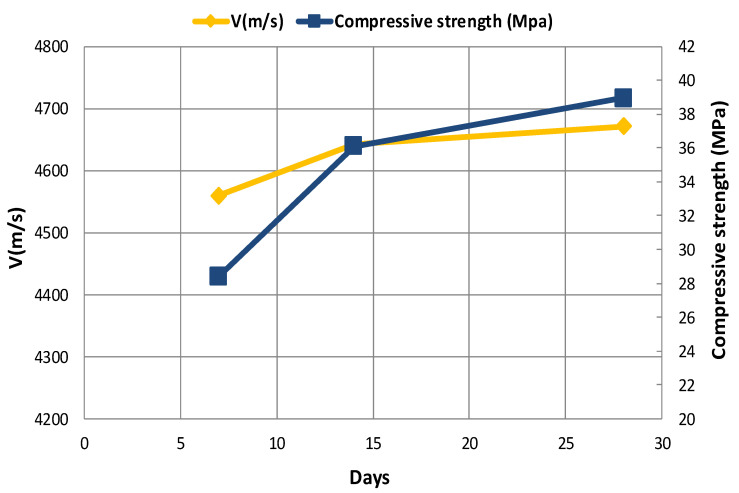
Comparison of UPV and compressive strength at 5% replacement of sand (S-J-S).

The relationship between the compressive strength and the UPV of concrete specimens versus curing time is shown in Figure 15, Figure 16 and Figure 17. The change in the compressive strength and UPV of concrete as a result of aging is noticeable in the control specimen and those specimens containing coal waste at 5% of aggregates volume. According to the figures, the compressive strength and UPV of the specimens demonstrate an almost equal increasing trend with increasing the age of the concrete. Therefore, using UPV as a non-destructive test method, the trend of strength variation can be well estimated as a curing time function. This fact implies that the compressive strength increases with increasing age of concrete specimens. Besides, due to the lower extent of porosity, and thus greater integrity of concrete, the UPV of concrete specimens is increased analogous to the compressive strength. Additionally, the results indicate that the pulse velocity is higher than the compressive strength at the early ages, i.e., before 14 days, the value of which is lower thereafter.

### 3.6. Relationship between Pulse Transmission Speed and Compressive Strength

**Figure 18:**
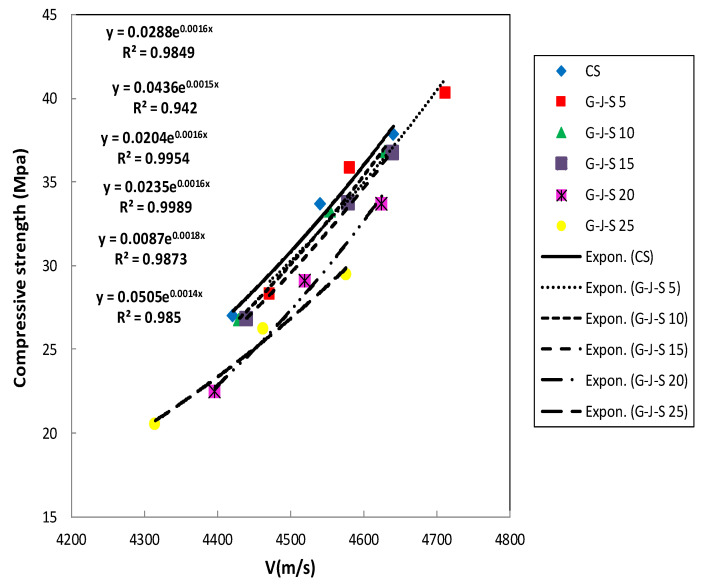
Relationship between UPV and compressive strength in the G-J-S group.

**Figure 19:**
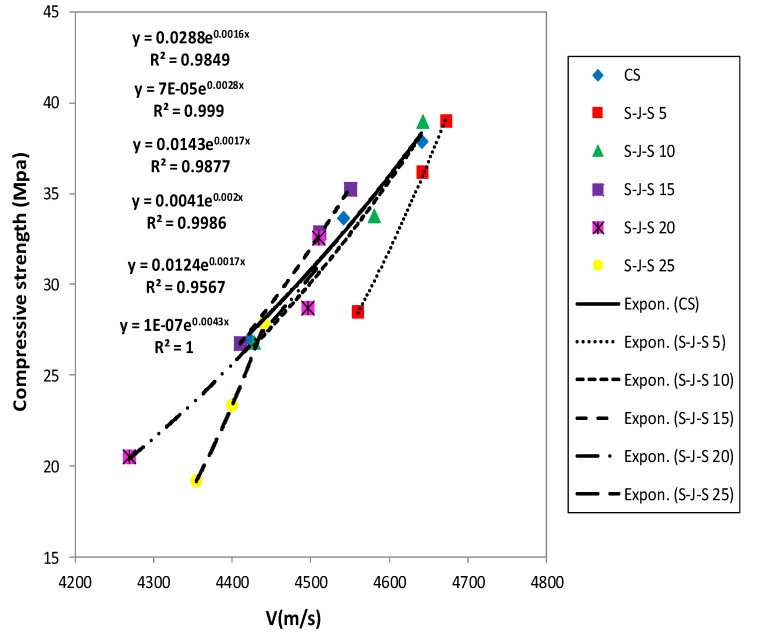
Relationship between UPV and compressive strength in the S-J-S group.

When UPV is used to characterize the concrete compressive strength, no specific relationship can be established between them. However, the elastic modulus of concrete is correlated with the compressive strength. Besides, the UPV is connected with the modulus of elasticity and density of concrete. Thereby, a priori motive to investigate the concrete compressive strength based on UPV can be discerned. Several researchers [18], [20], [21] have corroborated that the compressive strength is related to UPV in the form of an exponential function expressed by Equation (1), which also applies to concrete specimens containing waste materials [5,29,30]:

(1) $$f_{c}^{\prime }=A\;e^{\left(BV\right)}$$

in which $f_{c}^{\prime }$ and V, respectively, denote the compressive strength and UPV. Furthermore, A and B are the empirical constants. Correspondingly, an exponential curve was plotted between the data set using the nonlinear regression analysis. Figure 18 and Figure 19 represent the appropriate exponential functions in developing the relationship between UPV and compressive strength for the control specimen and those containing coal waste aggregates without considering the age of concrete. The coefficients of determination (R^2^) are also incorporated in these figures for the entire specimens. The closer this coefficient is to 1, the lower the scatter will be. Table 6 summarizes the empirical constants of A and B, and the regression coefficients (R^2^), for all mix designs.

**Figure 20:**
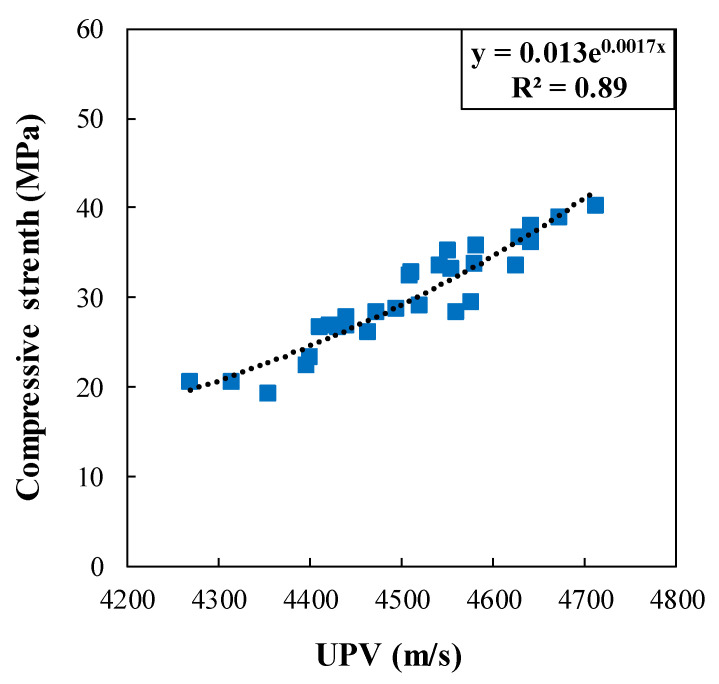
Experimental relationship between compressive strength and UPV together with test data for all concrete specimens.

Nonlinear regression analysis, including all test results of concrete specimens containing coal waste at different ages, was performed in order to provide a generic relationship between the compressive strength and the UPV, as expressed by Equation (2). It can be seen in Figure 20 that this equation has an acceptable coefficient of determination R^2^ = 0.89.

(2) $$f_{c}^{\prime }=0.013e^{0.0017\;V}$$

**Figure 21:**
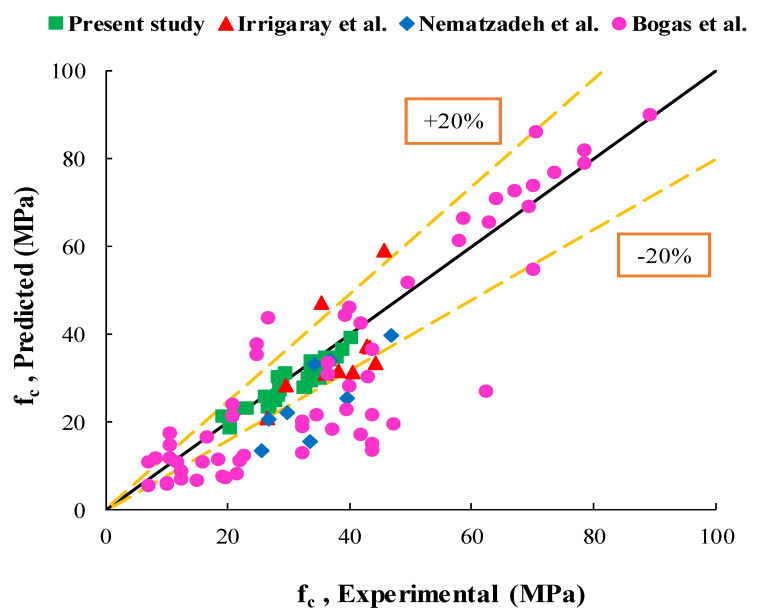
Comparison of the proposed model with experimental values of other researchers in literature and the present study.

To further assess and validate the proposed model, the compressive strength prediction results were compared in Figure 21 with other experimental research in the literature (Irrigaray et al. [31], Nematzadeh et al. [32], and Bogas et al. [18]). As shown in Figure 21, there is an approximately good accord between the results of compressive strength in the proposed relationship with the experimental results of other researchers so that the results of Irrigaray et al., compared to the proposed model, are closer to the experimental results of the present study. In addition, it is worth noting that Equation (2) was obtained by the nonlinear regression analysis.

## 4. Conclusions

In this study, the compressive strength of concrete containing coal waste aggregates was estimated using the non-destructive ultrasonic pulse velocity approach. For this purpose, concrete specimens were made in 11 experimental groups, and the parameters of compressive strength and UPV were investigated at different ages of concrete with different volume replacement levels of coarse and fine aggregates with coal waste. The results of this research are as follows:

1. The UPV in concrete specimens is reduced significantly as the replacement level of coarse and fine aggregates with coal waste is increased. However, UPV is increased at 5% substitution of coal waste.
2. The compressive strength of specimens is improved as 5 vol.% of natural aggregates is replaced with coal waste. However, further addition of coal decreases the compressive strength at different ages.
3. As the age of concrete specimens increases, the UPV gains value. In this respect, the maximum pulse velocity corresponds to the 28-day specimen with 5% of coal waste replacing coarse aggregates. In contrast, the minimum velocity belongs to the 7-day specimen, with 20% of fine coal waste aggregates.
4. The maximum compressive strength for the 28-day specimen with a replacement level of 5% of coarse coal waste is 40.3 MPa, which is approximately 6% higher than the control specimen at the same age. On the contrary, the lowest compressive strength for the 7-day specimen is 19.2 MPa with a 25% replacement of fine coal waste, indicating an approximate reduction of 29% compared to the control specimen at that age.
5. The exponential relationship between the compressive strength and the transmission speed of ultrasonic pulses, obtained in this experiment, shows the acceptable regression analysis results. Therefore, a generic exponential relationship between the compressive strength and UPV was proposed for the entire concrete specimen results with different volume replacement levels of coal waste at different ages, which correlates well with the test results.

## Figures and Tables

**Table 1:** Properties of natural aggregates.

Aggregate	Sand	Gravel
Specific gravity (g/cm^3^)	2.76	2.5
Unit weight (g/cm^3^)	1.73	1.57
Moisture content (%)	0.3	0.14
Moisture of saturated surface dry (%)	0.5	0.4
Fines modulus (FM)	2.92	-
Sand equivalent value (SE) (%)	82	-

**Table 2:** Chemical specifications of coal waste.

Items	SiO_2_	AL_2_O_3_	Fe_2_O_3_	MgO	CaO	P_2_O_5_–P_2_O_3_	Na_2_O	K_2_O	MnO	TiO_2_	L.O.I
Untreated Coal waste	37.8	13.14	2.85	0.73	0.76	0.27	0.28	2.02	0.02	1.17	40.96

**Table 3:** Properties of cement.

Chemical Properties	%
SiO_2_	21.9
Al_2_O_3_	4.86
Fe_2_O_3_	3.3
CaO	63.32
MgO	1.15
SO_3_	2.1
Loss on ignition	2.4
**Physical Properties**	
Specific gravity	3.15
Specific surface (m^2^/gr)	0.305
Initial setting time (min)	140
Final setting time (min)	190
**Mechanical Properties**	
Compressive strength (MPa)	18.14 (3 days)
	28.93 (7 days)
	37.17 (28 days)

**Table 4:** Concrete mix proportions.

Mix No.	Mixture ID	UCW (%)	W/C	Water	Cement	Coarse Agg.	Fine Agg.	UCW	Slump(mm)
				(Kg/m^3^)					
1	CS	0	0.55	215	391	854	855	0	80
2	G-J-S	5	0.55	215	391	811.3	855	32.04	80
3		10	0.55	215	391	768.6	855	60.70	70
4		15	0.55	215	391	725.9	855	86.00	75
5		20	0.55	215	391	683.2	855	107.92	80
6		25	0.55	215	391	640.5	855	126.47	82
7	S-J-S	5	0.55	215	391	854	812.25	29.11	70
8		10	0.55	215	391	854	769.5	55.15	70
9		15	0.55	215	391	854	726.75	78.14	68
10		20	0.55	215	391	854	684	98.05	60
11		25	0.55	215	391	854	641.25	114.91	58

UCW denotes the untreated coal waste, S-J-S designates the sand jig specimen (fine recycled aggregates), and G-J-S represents the gravel jig specimen (coarse recycled aggregates).

**Table 5:** Test results of ultrasonic pulse velocity (UPV) and compressive strength of concrete specimens.

Mix No.	Group	Coal Waste	7 Days		14 Days		28 Days	
			$f_{c}^{\prime }\;(\mathrm{MPa})$	V(m/s)	$f_{c}^{\prime }\;(\mathrm{MPa})$	V(m/s)	$f_{c}^{\prime }\;(\mathrm{MPa})$	V(m/s)
1	CS	0	26.96	4421	33.66	4541.2	37.85	4641.2
2	G-J-S	5	28.28	4472.4	35.8	4581.4	40.28	4712.4
3		10	26.71	4430	33.24	4554.2	36.8	4629.5
4		15	26.82	4440	33.75	4580	36.74	4640
5		20	22.42	4396.6	29.07	4520	33.65	4624.7
6		25	20.5	4315	26.17	4463.5	29.43	4576.8
7	S-J-S	5	28.41	4560	36.12	4641.7	38.94	4671.7
8		10	26.82	4428	33.72	4580.4	37.98	4641.4
9		15	26.71	4410	32.86	4510.3	35.23	4550.5
10		20	20.47	4354	28.71	4495.1	32.51	4509.3
11		25	19.18	4268.9	23.33	4400	27.81	4440.4

**Table 6:** Exact values of empirical constants and correlation coefficients in UPV–compressive strength relationship.

Mix no	Group	Coal Waste (%)	A	B	R^2^
1	CS	0	0.0288	0.0016	0.9849
2	G-J-S	5	0.0436	0.0015	0.942
3		10	0.0204	0.0016	0.9954
4		15	0.0235	0.0016	0.9989
5		20	0.0087	0.0018	0.9873
6		25	0.0505	0.0014	0.985
7	S-J-S	5	0.00007	0.0028	0.999
8		10	0.0143	0.0017	0.9877
9		15	0.0041	0.002	0.9986
10		20	0.0124	0.0017	0.9567
11		25	0.0000001	0.0043	1

## Data Availability

The data used to support the findings of this study are available from the corresponding author by request.
